# The Interplay Between Economic Status and Attractiveness, and the Importance of Attire in Mate Choice Judgments

**DOI:** 10.3389/fpsyg.2019.00462

**Published:** 2019-03-21

**Authors:** Amany Gouda-Vossos, Robert C. Brooks, Barnaby J. W. Dixson

**Affiliations:** ^1^Evolution and Ecology Research Centre, School of Biological, Earth and Environmental Sciences, The University of New South Wales, Sydney, NSW, Australia; ^2^School of Psychology, The University of Queensland, Brisbane, QLD, Australia

**Keywords:** sex difference, status, attire, attractiveness, mate choice copying, economics

## Abstract

Desirable characteristics of “opposite sex others,” such as physical attractiveness and economic status, can influence how individuals are judged, and this is different for men and women. However, under various social contexts where cues of higher or lower economic status is suggested, sex differences in judgments related to mate choice have not been fully explored. In two studies, ratings of economic status and attractiveness were quantified for male and female targets that were presented under various social contexts. Study 1 assessed judgments (*n* = 1,359) of images of nine male and nine female targets in different sized groups containing only opposite-sex others (i.e., group size). While we found no significant effects of group size on male and female attractiveness, target female economic status increased when surrounded by two or more men. An *ad hoc* analysis controlling for the attire of the targets (business or casual) found that the association between target female economic status and group size occurred when females were in business attire. Study 2 investigates this effect further by presenting images of 12 males and 12 females, in higher and lower status attire (i.e., business and casual clothing) and measured judgments of attractiveness and economic status among women and men (*n* = 1,038). Consistent with the results of Study 1, female economic status was only affected when women were in business attire. However, female economic status decreased when in the presence of other men in business attire. There were no sex differences in judgments of economic status when judging stimuli in casual attire. Additionally, negative associations between attractiveness and economic status were found for males presented in casual attire. We discuss these results in the light of evolutionary sexual conflict theory by demonstrating how the asymmetrical importance of status between men and women can influence mate choice judgments.

## General Introduction

Evolved mate preferences often target attributes that signal dimensions of reproductive health (Buss and Schmitt, 1993; Puts, 2010). In women, age-related physical cues such as feminine facial shape, breast morphology, and an hourglass distribution of body fat are attractive to men (Jasieńska et al., 2004; Singh et al., 2010; Dixson et al., 2011, 2015; Marcinkowska et al., 2014), ostensibly because they signal fecundability. In men, muscularity, vocal pitch, and facial masculinity provide information regarding health, age, social status, dominance, and formidability, that enhance mating success (Archer, 2009; Puts, 2010; Hill et al., 2013; Dixson et al., 2014).

Judgments of physical attractiveness are also shaped by factors other than physical attributes (Dixson, 2019; Luoto, 2019). For instance, men are more likely to be rated as more physically attractive when they are presented with high status cues such as an expensive car (Dunn and Searle, 2010; Shuler and McCord, 2010) or an upscale apartment (Dunn and Hill, 2014). While these cues may not influence ratings of women’s physical attractiveness, high status may drive intersexual competition between women (Wang and Griskevicius, 2013). Judgments of physical attractiveness also increase with the addition of other people, an effect known as “mate choice copying” (Waynforth, 2007). Men are more likely to be rated as more attractive and to have higher economic status when in the presence of women, whereas mate choice copying effects are negligible when women are in the presence of men (Gouda-Vossos et al., 2016, 2018).

The associations between sex, economic status, and physical attractiveness may reflect evolved sex differences in mate choice (Gouda-Vossos et al., 2018). Throughout human evolution, resource acquisition positively influenced male reproductive success (Low, 1990, 2000; Smith, 2004), so that sexual selection may have favored status seeking behavior in men (Betzig, 1986; Dixson, 2016; Von Rueden and Jaeggi, 2016). An association between men’s status and reproductive success has been reported across many small-scale (Von Rueden and Jaeggi, 2016) and several industrialized societies (Li et al., 2002; Hopcroft, 2006), which may have implications regarding the formation of social perceptions of women and men. For instance, participants judged female economic status comparatively lower than that of the males they were presented alongside (Gouda-Vossos et al., 2016). Unlike men, women’s success can be judged negatively, as high status or successful women are more frequently derogated (Heilman et al., 2004), particularly when dressed in short skirts or shirts displaying cleavage (Glick et al., 2005; Howlett et al., 2015). Conversely, physical attractiveness is beneficial to women as attractive individuals receive favorable treatment (Rosenblat, 2008) and are more likely to find jobs and get promoted (Hamermesh and Biddle, 1993; Pfann et al., 2000), which benefits women more than men within employment scenarios (Busetta et al., 2013). However, the interplay between physical attractiveness, economic status, and attire in mixed social contexts that are more comparable to real-world scenarios has yet to be explored.

The current research assesses how modifiable cues of economic status influence judgments of an individual’s physical attractiveness and economic status. Previous studies reported that high status men were more likely to receive respect and praise compared to women in high status roles (Forsythe, 1990; Brase and Guy, 2004). Studies have also shown that high status women within mixed sex groups were just as likely as men to attain leadership positions (Goktepe and Schneier, 1989). However, high status women were judged to be less attractive and approachable than high status men (Howlett et al., 2013). Additionally, ratings of women’s economic status are lower relative to men they are presented alongside (Gouda-Vossos et al., 2016). However, whether this association persists when women are presented as higher in economic status than men or if physical attractiveness influences ratings of economic status remains unknown.

Based on evolutionary theories regarding the importance of status in male reproductive success (Hopcroft, 2006; Von Rueden et al., 2010) and mate choice copying theory (Waynforth, 2007; Gouda-Vossos et al., 2018), we predicted that cues of higher social status should have a stronger positive effect on ratings of economic status and physical attractiveness in men than in women. We also predicted that women’s economic status would be rated lower than the men they were presented alongside, even if women appeared to be higher in economic status than men (Gouda-Vossos et al., 2016). We conducted two studies, both of which manipulated economic status via clothing in male and female stimuli and measured participant attractiveness and monetary earnings ratings of the stimuli. We first tested the effects of the presence of “opposite sex others” by manipulating mixed sex group sizes (Study 1). Based on the results of Study 1, we designed Study 2 wherein various forms of attire were used to manipulate social status, including the presence and absence of “opposite sex others” in various forms of attire.

## Study 1: Judgements of Attractiveness and Economic Status Within Mixed-Sex Groups

Dynamics within groups can vary depending on the size of social groups and the distributions of gender therein. All-male groups tend to be more aggressive and competitive toward other group members than all-female groups (Schopler et al., 2001). Additionally, all-male groups form more stable hierarchies faster than all-female groups (Anderson et al., 2001) and are more likely to collaborate intra-sexually when an outside threat is present (Vugt et al., 2007). Sharing cooperatively produced resources is also an important factor within collective groups (Mangel, 1990; Melis and Semmann, 2010) and the dynamics within groups can vary if some members are more likely to obtain larger portions of resources (relative to other members) and subsequently gain direct benefits (Williams, 2002). Among men, resource acquisition and holding potential enhance mating opportunities and mating success (Betzig, 1986; Von Rueden et al., 2010). As a result, expectations and opportunities vary between men and women within mixed-sex groups (Eagly and Johnson, 1990). Mate choice copying studies have also found that men are judged to be more physically attractive when presented within a group of women, while women presented alongside men are not (Milonoff et al., 2007; Hill and Buss, 2008; Dunn and Doria, 2010).

Behaviors within groups may be driven by similar mechanisms associated with sex differences in mate choice, especially in reference to associations between status (social or economic) and physical attractiveness. In men, cues of social status, dominance, and formidability that enhance male physical attractiveness (Hill and Buss, 2008; Archer, 2009; Puts, 2010; Dixson et al., 2017, 2019) and mating success (Hill et al., 2013; Kordsmeyer et al., 2018) may also predict assertiveness and group leadership (Anderson et al., 2001; Geniole et al., 2015). While cues of status may also predict the emergence of female leaders in groups (Anderson et al., 2001) they may not augment women’s physical attractiveness and mating success (Puts, 2010). To test the effects of social group size on ratings of male and female attractiveness and economic status, we presented images of women and men in the presence of social groups varying in the number of opposite sex targets. Thus, each male and female was rated alone and again alongside opposite sex targets in increments of 1, 2, and 4 additional opposite sex individuals.

### Materials and Methods

#### Participants

Participants were recruited via Facebook, Twitter, and internal student email lists within the research institution, resulting in 1359 participants in total. All participants were over 18 years old and were not aware of the purpose of the study. Each participant provided details of their biological sex, age, and sexual orientation using a Kinsey Scale (Kinsey et al., 1948, 1953). As sexual orientation impacts on judgments of attractiveness of opposite sex targets (Petterson et al., 2015, 2016, 2018; Valentova et al., 2017), only participants who were heterosexual or bisexual were retained in the analyses (i.e., Kinsey scale 0–3). The final analysis included 569 (women = 494; men = 75) participants who completed surveys including target males and 598 (women = 357; men = 241) who completed the survey’s including target females. The age range of participants was 29.04 years ± 9.4. The majority of participants listed their country of origin as Australia (38.7%), followed by USA (20%), then UK (17.7%). The majority identified as North Western European, British, or Irish (51.2%), followed by European Mixed Race (13.8%), then Southern European (3.8%) with 8% stating they “did not wish to report ethnicity.”

#### Stimuli

Images of nine male and nine female targets surrounded by four members of the opposite sex (females and males, respectively) were chosen from a stock photo website^1^. This resulted in a total of 18 original images. Each target was presented in four group size conditions [alone, one opposite sex, two opposite sex, and four opposite sex others; for examples, see Electronic Supplementary Material S1 (ESM 1)]. Overall, 72 images were constructed and used in this study, with the target pose and facial expression identical between treatments. The targets ages ranged from 22 to 56 years (males: mean = 40, *SD* ±12.3, females: mean = 38, *SD* ±12.7). All images were professionally taken under standardized lighting and filters. Photographs were taken in workplaces and casual settings with positions of targets and opposite sex others varying from image to image.

#### Procedure

Experiments were conducted on-line via www.socialsci.com. Each participant entering the study was randomly assigned to one of four experiments in which they rated either male or female targets for either attractiveness or monetary earning (i.e., economic status). The number of participants for each experiment was as follows: Attractiveness/target female: 202 women, 150 men; Earnings/target female: 155 women, 91 men; Attractiveness/target males: 259 women, 39 men; Earnings/target males: 235 women, 36 men. The study employed a “Within Target – Between Treatment” design where participants saw all nine targets in random order with the treatment (target alone, one opposite sex other, two opposite sex others, and four opposite sex others) for each target drawn at random. Similar designs have been used in past research on physical attractiveness (Janif et al., 2014, 2015; Brooks et al., 2015; Dixson et al., 2016). This research was approved by the University of New South Wales Human Research Ethics Advisory Board (Psychology) (HREAP 1880).

Participants were informed that they would be shown a range of images of people. In each image, the target was indicated with an arrow. If assigned to rate physical attractiveness, participants were asked to rate each target using a percentile scale from 0 to 100 where “50” indicated that the individual is more physically attractive than 50% of other individuals of the same sex (i.e., of median attractiveness). If rating economic status, participants were asked to rate each target using a percentile scale from 0 to 100 where “50” indicated the individual earns more than 50% of other same sex individuals in full time work (i.e., median income in full-time work).

#### Analysis

Multilevel modeling was used where data were organized so that each row represented one participants rating of one target in one treatment. Using the statistical software SPSS, separate general linear mixed models (MLMs) were fitted for the two dependant variables (physical attractiveness or economic status). In each of these models, Model ID was a repeated-measures factor, Participant ID was a random factor. Participant sex and Group Size (alone, +1 opposite sex individual, +2 opposite sex individual, and +4 opposite sex individuals) were included as fixed factors. SPSS does not calculate effect sizes for mixed models. Thus, we calculated approximate effect sizes as partial Eta-squared, from the *F*-test and degrees of freedom, although this practice has not been formally validated for multi-level models. When interpreting effect sizes, by convention, effects of 0.2, 0.5, and 0.8 are interpreted as small, medium, and large effect sizes, respectively.

### Results

#### The Effect of Group Size on Male and Female Attractiveness and Economic Status

**FIGURE 1:**
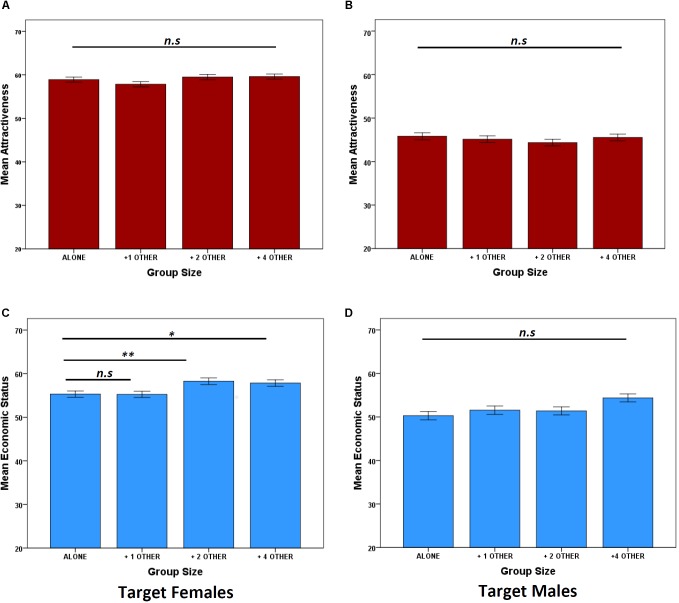
Data are mean ratings of attractiveness **(A,B)** and economic status **(C,D)** ratings (±1 SEM) split by four group size (alone, 1,2,4 opposite sex others). ^∗^*P* < 0.05, ^∗∗^*P* < 0.01, determined by *post hoc* least significance difference tests.

There were no significant main effect or interactions involving Group Size; suggesting no differences in the ratings of attractiveness across treatment (Figure 1A,B and Table 1a). The significant main effects of participant sex on ratings of target females were due to women rating target females as more attractive (mean = 59.85, *SE* ±0.391) than men (mean = 58.66, *SE* ±0.456).

**Table 1:** MLMs for target male and female rated attractiveness and economic status.

	Target female			Target males				
		
	*df*	*F*	*P*-value	$\eta_{p}^{2}$	*df*	*F*	*P*-value	$\eta_{p}^{2}$
**(a) Attractiveness**								
Akaike Information Criterion	**26,858.21**				**23,691.96**			
Group size	3, 3,074.69	1.74	0.156	0.002	3, 2,569.39	0.12	0.951	<0.001
Participant sex	1, 3,085.56	3.90	**0.048**	0.002	1, 2,597.86	1.11	0.291	<0.001
Group size ^∗^ participant sex	3, 3,074.69	0.21	0.893	<0.001	3, 2,569.39	0.97	0.406	<0.001
**(b) Economic status**								
Akaike Information Criterion	**18,833.71**				**22,008.02**			
Group size	3, 2,084.79	4.50	**0.004**	0.006	3, 1,696.49	1.51	0.210	0.003
Participant sex	1, 2,109.86	1.66	0.198	<0.001	1, 1,730.98	3.06	0.080	0.002
Group size ^∗^ participant sex	3, 2,084.79	0.35	0.788	<0.001	3, 1,696.49	0.23	0.874	<0.001


Like the results for attractiveness, male ratings of economic status were not affected by Group Size as there was no significant main effect or interactions with participant sex (Figure 1C and Table 1b). However, ratings for target females revealed a significant main effect of Group Size (Table 1b) as female economic status increased incrementally with the addition of two and four males (Figure 1D). There was no significant Participant Sex × Group Size interaction, suggesting that men and women were rating target females similarly (Table 1b).

#### The Effect of Attire and Group Size on Target Attractiveness and Economic Status; an *ad hoc* Analysis

Images included targets in either casual or business attire, which may have affected ratings. To test this, targets were classified as wearing either business or casual attire using methods from Forsythe (1990). Business attire referred to dark, angular, traditional business suits whereas casual attire referred to light, informal, everyday wear. There were four business and five casual attired target males and five business and four casual attired target females. Full analysis can be found in Electronic Supplementary Materials S2, S3 (ESM 2: Male and Female Attractiveness and ESM 3: Male and Female Economic Status).

Another series of MLMs were conducted, with attire included as a fixed factor. We found no effects of Attire on target male attractiveness (*F*_1,2555_ = 0.851, *P* = 0.356, $\eta_{p}^{2}$ = 0.00033), Group Size (*F*_3,2555_ = 0.091, *P* = 0.965, $\eta_{p}^{2}$ = <0.001), and Participant Sex (*F*_1,2555_ = 0.961, *P* = 0.339, $\eta_{p}^{2}$ = <0.001). The effect sizes for all main effects were small for male attractiveness (i.e., less than 0.2), suggesting that both Attire and Group Size do not strongly impact on male attractiveness.

There was a main effect of Attire on target male economic status (*F*_1,1877_ = 533.83, *P* < 0.001, $\eta_{p}^{2}$ = 0.220), but not Group Size (*F*_3,1853_ = 1.408, *P* = 0.239, $\eta_{p}^{2}$ = 0.002), or Participant Sex (*F*_1,1877_ = 2.156, *P* = 0.142, $\eta_{p}^{2}$ = 0.001). Both men and women rated male economic status higher when in business attire (mean = 65.32, *SE*±0.713) than when in casual attire (mean = 39.43, *SE* ±0.865). We did not find mate choice copying effects, which suggest that the type of attire men were wearing influences ratings of target males more than the presence of other females.

There was a main effect of Attire on attractiveness ratings of target females (*F*_1,2963_ = 68.13, *P* < 0.001, $\eta_{p}^{2}$ = 0.023) but no main effect of Group Size (*F*_3,2952_ = 1.937, *P* = 0.121, $\eta_{p}^{2}$ = 0.002) or Participant Sex (*F*_1,2963_ = 2.982, *P* = 0.084, $\eta_{p}^{2}$ = 0.010). Target females were rated as more attractive when in business (mean = 61.46, *SE* ±0.404) than Casual Attire (mean = 56.52, *SE* ±0.442), although the effect size was small (i.e., below 0.2) and comparable to Group Size, suggesting that the impact of Attire on female attractiveness is small.

**FIGURE 2:**
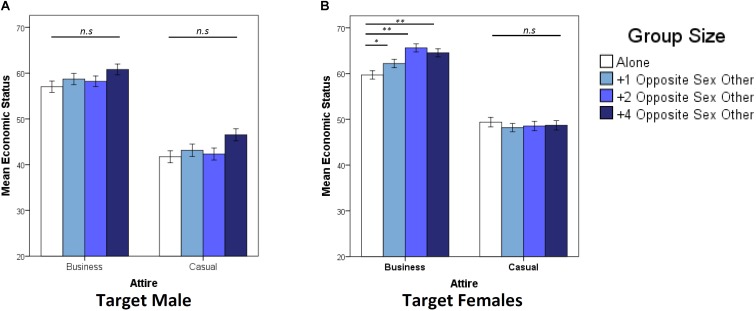
Data are mean economic status ratings (±1 SEM) of target males **(A)** and females **(B)**, split by target attire (business/casual) for four group size treatments (alone, 1,2,4 opposite sex others). ^∗^*P* < 0.05, ^∗∗^*P* < 0.01, ^∗∗∗^*P* < 0.001, determined by *post hoc* least significance difference tests.

Ratings of female earnings were also significantly affected by Attire (*F*_1,2075_ = 356.54, *P* < 0.001, $\eta_{p}^{2}$ = 0.150) and Group Size (*F*_2,2057_ = 3.181, *P* = 0.023, $\eta_{p}^{2}$ = 0.005), although both effect sizes were small (i.e., below 0.2). A significant Attire × Participant Sex interaction (*F*_1,2057_ = 18.68, *P* < 0.001, $\eta_{p}^{2}$ = 0.009) occurred due to women rating females higher in economic status when in business attire than men (women mean = 63.57, *SE* ±0.566; men mean = 61.6, *SE* ±0.740; *P* = 0.043) and lower when in casual attire than men (women mean = 47.58, *SE* ±0.622; men mean = 51.89, *SE*±0.785; *P* < 0.001). There was also a significant Attire × Group Size interaction (*F*_1,2057_ = 6.369, *P* < 0.001, $\eta_{p}^{2}$ = 0.009), which reflects that ratings of female economic status increased incrementally with the addition of two and four males when females were presented in business but not casual attire (Figure 2A,B). This suggests that the original results of female economic status were likely driven by the responses toward target females in business attire, as opposed to casual attire.

In order to determine the model of best fit, we used the Akaike Information Criterion (AIC). We monitored the AIC between the original models and the *ad hoc* analysis. If AIC changes downward by more than 2 units, the model is significantly better (Bozdogan, 1987). Using both the level of significance of interactions and the AIC allows us to test the validity of different restrictions of a model and to choose a model with the smallest probability of rejection to be the best fitting model as opposed to choosing based on *a priori* ground (Bozdogan, 1987). Across all four *ad hoc* models, the AIC was significantly lower than the original models constructed (Female Attractiveness, Original AIC: 26858.21, *ad hoc* AIC: 26,768.36; Male Attractiveness, Original AIC: 23,691.96, *ad hoc* AIC: 23,657.82; Female Earnings, Original AIC: 18,833.71, *ad hoc* AIC: 18,405.63; Male Earnings, Original AIC: 22,008.02, *ad hoc* AIC: 21,434.76). This suggests that entering Attire as a fixed factor improves all the models.

### Discussion

Contrary to our predictions, ratings of attractiveness for target male and females were not influenced by social group size. Further, the economic status of males was unaffected when in the presence of an opposite sex other (i.e., female). Whereas the addition of opposite sex others positively influenced the economic status of target females. Previous studies have found that woman’s task proficiency (Balkwell and Berger, 1996), economic status (Gouda-Vossos et al., 2016), and social status (Eagly and Karau, 2002) were rated lower than the men they were compared with, suggesting that the mere presence of a man can lower perceptions of women’s status within an economic hierarchy. However, the type of attire women wear may reduce these effects as women dressed in more masculine attire (i.e., traditional business attire) are seen as having better managerial characteristics (Forsythe et al., 1984, 1985) are more likely to get hired for leadership positions (Forsythe, 1990), and are just as likely as men to emerge as leaders within a mixed sex group (Goktepe and Schneier, 1989). Taken together with the results of the current studies, women’s perceived economic status appears to be heavily influenced by high status and masculine cues such as business attire and the number of men within their immediate presence.

However, we are limited in making these assumptions regarding attire, as the numbers of targets used were too small after separation for *ad hoc* analysis (i.e., four business and five casual attired target males and five business and four casual attired target females). Further, the position of the target in each photo was not randomized or controlled and therefore we could not conclude whether participants perceived targets as leaders, which could have influenced ratings of attractiveness and economic status. It is also possible that even though female economic status increased with the addition of “male others,” ratings of female status may still be made relative to men. Unfortunately, we did not obtain ratings of earnings and attractiveness of the male “opposite sex others” to confirm this. Thus, we designed a second study focused on the impact of attire of various social status (business/casual) and measured effects of male and female attractiveness and economic status in targets presented individually and when paired (Study 2).

## Study 2: The Effects of Attire on Men and Women

Attire communicates information relating to identity, social status, and position within a hierarchy (Roach-Higgins and Eicher, 1992). Molloy and Potter (1975) suggested that attire may play a pivotal role in judgments of an individual’s credibility, likeability, interpersonal attractiveness, and dominance. Bassett et al. (1979) found that high status clothing positively influenced judgments of credibility. However, females were rated lower than males across all four measurements that composed credibility (i.e., potency, character, composure, and competence). Unlike men, women’s success can be judged negatively, as high status or successful women are more frequently derogated (Heilman et al., 2004) and are judged more negatively when dressed in short skirts (Glick et al., 2005; Howlett et al., 2015). However, physically attractive women receive better treatment (Rosenblat, 2008), are more likely to find employment, and are more likely to get promoted (Hamermesh and Biddle, 1993; Pfann et al., 2000), which may not be the case among men (Busetta et al., 2013).

In Study 2, we measured associations between rated physical attractiveness and economic status in male and female targets in different attires. We also assessed if the presence of opposite sex others in various attire influenced judgments of male and female targets. Based on mate choice copying theories (Waynforth, 2007; Gouda-Vossos et al., 2018), we hypothesized that males would attain high ratings of physical attractiveness and economic status when presented in high status attire (i.e., business attire) regardless of the attire the opposite sex other. As economic status may not have had strong effects on female reproductive success during human evolution (Betzig, 1986), we did not predict a positive association between attractiveness and economic status with target females. Based on the results of Study 1, we predicted that female economic status will be limited to the ratings of males when in casual but not business attire.

### Materials and Methods

#### Participants

A total of 1,035 participants were recruited. All participants were over 18 years old and were not aware of the purpose of the study. Each participant provided details of their biological sex, age, and sexual orientation using the Kinsey Scale (Kinsey et al., 1948, 1953). Participants received $1US. As in Study 1, only participants who were heterosexual or bisexual were retained in the analyses (i.e., Kinsey scale 0–3). A total of 459 females and 578 males (Age 32 ± 10.5) were included in the final analysis. The majority of participants listed their country of origin as United States (82.4%), followed by Southern Europe (4.4%), then Australia (3.4%). The majority identified as ethnically North Western European, British, or Irish (40.4%), followed by European Mixed Race (17.2%), Southern European (7.2%), and 10% elected not to state their ethnicity.

#### Stimuli

**FIGURE 3:**
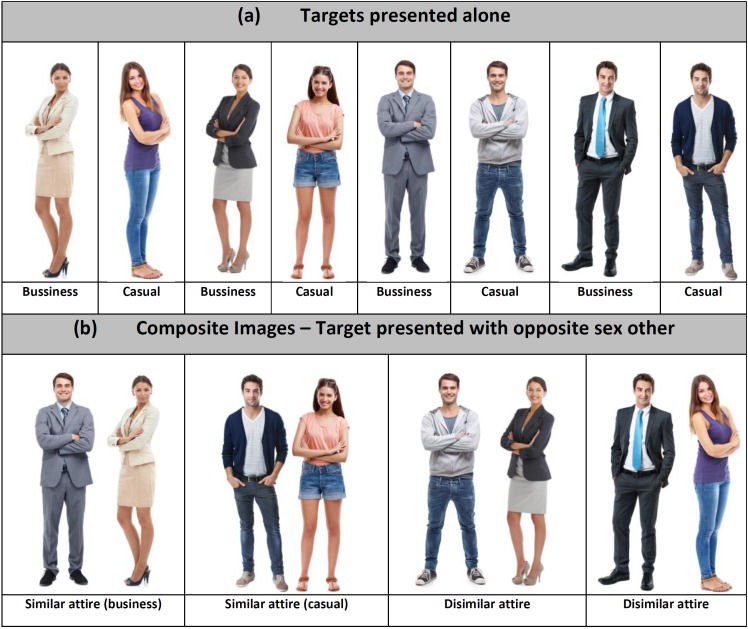
Example images used for targets presented alone **(A)** and with opposite sex others **(B)**. Film Media courtesy of Yuri Arcurs Photography Aps, Used by Permission.

Full body, color photographs of 12 male and 12 female targets were obtained from a stock photo website^2^. All photographs were taken using standardized lighting and filters and were on a white background. Two sets of images for each male and female target were obtained (i.e., either in casual attire or business attire), comprised of 24 male and 24 female target images. We then created composite images, where each target (in both business and casual attire) was paired with opposite sex others (six in business and six in casual attire), resulting in a total of 144 composite images for male targets and 144 composite images for female targets (Figure 3). Business attire included suits, collared shirts, and pencil skirts (for females not wearing pant suits). Casual attire included t-shirts, jeans, shorts, or skirts (Figure 3). PeopleImages.com collects information on the targets they recruit, including biological sex, age, and ethnicity. The targets ages ranged from 20 to 30 years (Males Target mean = 25, *SD* ±4.8 years; Females Target Mean = 24, *SD* ±4.3) and the majority were Caucasian (66%) followed by multi-ethnic (16%), then African and Latino (9% each).

#### Procedure

Studies were conducted online using the SocSci platform^3^ and participants were recruited via MTurk. Participants each rated two batches of 12 images (24 in total). One batch of 12 images included either male or female targets alone. The other batch included male or female targets with opposite sex others. The order of the batches as well as the target sex was fully randomized, so that participants were presented with four possible combinations (i.e., female alone/male with other; female with other/male alone; male alone/female with other; and male with other/female alone). Within each batch, each target was drawn and shown once, in random order and either in casual or business attire (drawn at random with equal probability) (see Figure 3A,B for examples). Thus, each target was presented to the participant either alone or with an opposite sex other, in either business or casual attire. This design ensured participants saw all possible targets (male and female) in only one type of attire (i.e., Within Target – Between Treatment design). By designing the study in this manner, participants do not see the same target more than once in different scenarios, which minimizes possible carry-over effects and avoided participants deciphering the true nature of the study that previous studies have shown to influence ratings of targets (Chen, 2008). Experimental designs like this have been previously employed to test preferences for physical appearance (Janif et al., 2014, 2015; Brooks et al., 2015; Dixson et al., 2016). Participants rated targets using a sliding scale (from 0 to 100) provided below each image for physical attractiveness and economic status using the same scales as in Study 1. This research was approved by the University of New South Wales Human Research Ethics Advisory Board (HREA 155047).

#### Analysis

Using the statistical software, SPSS, we first tested the influence of individual sex and attire on ratings of attractiveness and economic status by focusing on the ratings of attractiveness and economic status of targets when presented alone. This allowed us to determine how target attire (business/casual) and target sex (female/male) influence these ratings. This 2 × 2 between-subject design (Sex of target – Male/Female) × (Attire of Target – business/casual) employed MANOVAs, with rated attractiveness and economic status as dependant variables.

We then assessed if the attire of the opposite sex other influenced male and female attractiveness and economic status by focusing on ratings of targets when presentenced with opposite sex others. This was a 2 × 2 × 2 × 2 between-subject design (Sex of target – Male/Female) × (Attire of TARGET – business/casual) × (Attire of OTHER – business/ casual) × (Participant Sex – Men/Women). We analyzed the results of targets when presented with an opposite sex other of varying attire (business/casual), using separate general linear mixed models (MLM) for ratings of physical attractiveness and economic status for each study. Target ID, Subject ID, and OtherID were included as random factors to specify the covariance structure for the residuals. Target Attire, Target Sex, Other Attire, and Participant Sex were fixed factors. All main effects and interactions were assessed.

### Results

#### Effects of Individual Attire and Sex on Attractiveness and Economic Status Ratings

**FIGURE 4:**
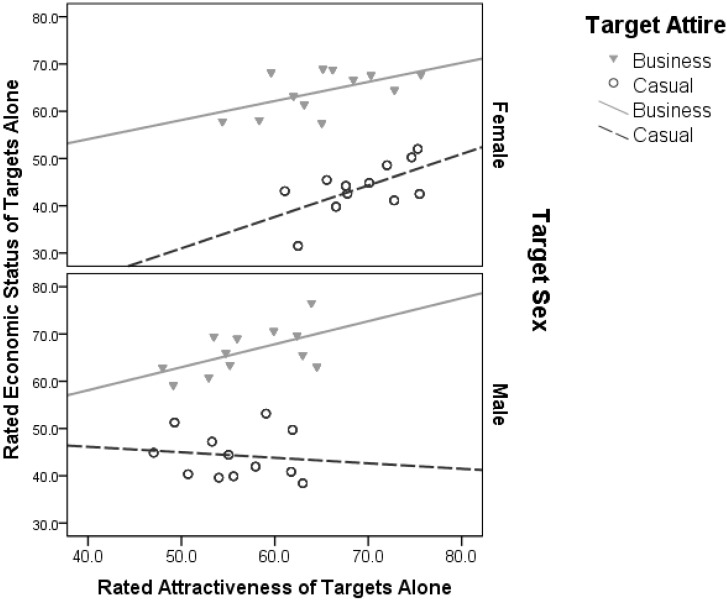
The association between rated attractiveness and economic status of target males and females when presented alone in business and casual attire.

The multivariate analysis revealed significant main effect of Target Sex, Target Attire, and their interaction (Table 2). For female targets, there were positive associations between Attractiveness and Economic status ratings when presented in both casual and business attire (Figure 4). In contrast, male targets were rated negatively for attractiveness and economic status when in casual attire (Figure 4). There was a significant Target Sex × Target Attire interaction (Table 2), which reflects target females were rated lower for economic status (target female mean = 54.03, *SD* ±11.27; target male = 55.33, *SD* ±12.04) but higher for attractiveness (target female = 67.178, *SD* ±5.33; target male = 56.32, *SD* ±5.27) than male targets. There was also a significant Target Sex × Target Attire interaction (Table 2), so that target females were rated as more attractive in casual attire (mean = 69.28, *SD* ±4.72) than business attire (mean = 65.08, *SD* ±5.91).

**Table 2:** MANOVA for rated economic status and attractiveness for targets presented alone.

Within subject effects												

	Target sex				Target attire				Target sex ^∗^ target attire			
					
Pillai’s trace	*F*	*df_n_*	*P*	$\eta_{p}^{2}$	*F*	*df_n_*	*P*	$\eta_{p}^{2}$	*F*	*df_n_*	*P*	$\eta_{p}^{2}$
MANOVA	202.22	2,283	**<0.001**	0.588	895.93	2,283	**<0.001**	0.864	9.39	2,283	**<0.001**	0.062
**Between subject effects**												
Economic status	5.358	1,284	**0**.**021**	0.019	1426.96	1,284	**<0.001**	0.834	2.15	1,284	**0.144**	0.008
Attractiveness	301.98	1,284	**<0.001**	0.515	5.706	1,284	**0.018**	0.020	18.76	1,284	**<0.001**	0.620


#### The Influence of Target Attire and the Attire of Opposite Sex Other on Target Attractiveness and Economic Status

**FIGURE 5:**
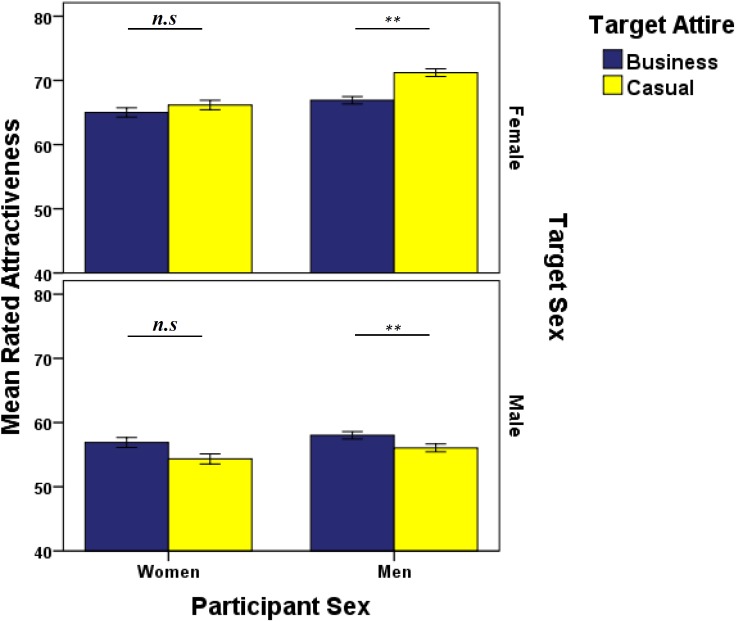
Data are mean attractiveness ratings (±1 SEM) from women and men (participant sex), split by target sex for two attire treatments (business/casual). ^∗∗^*P* < 0.01, determined by *post hoc* least significance difference tests.

There was a significant main effect of Target Sex (Table 3a), so that female targets were rated as more attractive than male targets (Target Female mean = 67.32, *SE* ±1.20; Target Male Mean = 56.32, *SE* ±1.16; *P* < 0.001). The Target Sex × Participant Sex interaction was not statistically significant (Table 3a). There was a significant Target Attire × Target Sex × Participant Sex interaction for attractiveness ratings that was driven by the ratings from men, who rated female attractiveness lower when in business attire than casual; and male attractiveness higher in business than casual (Table 3a and Figure 5). There were no significant effects due to women’s ratings (Figure 5).

**Table 3:** MLMs for target male and female rated attractiveness and economic status.

	(a) Rated attractiveness				(b) Rated economic status			
		
AIC without factors (random factors only)	54,248.21				51,332.19			
AIC including factors	54,073.10				49,135.69			
	*F*	*df*	*P*	$\eta_{p}^{2}$	*F*	*df*	*P*	$\eta_{p}^{2}$
Target attire	1.69	1,6056.64	0.193	<0.001	2600.64	1,5520.90	**<0**.**001**	0.320
Target sex	87.27	1,540.25	**<0**.**001**	0.139	9.68	1,489.30	**0**.**002**	0.019
Other attire	1.39	1,6050.47	0.238	<0.001	1.72	1,5561.22	0.189	<0.001
Participant sex	4.30	1,540.23	**0**.**039**	0.008	0.82	1,489.30	0.365	0.002
Target attire ^∗^ target sex	34.15	1,6054.93	**<0**.**001**	0.006	47.32	1,5520.88	**<0**.**001**	0.009
Target attire ^∗^ others attire	0.69	1,6045.44	0.405	<0.001	7.39	1,5529.43	**0**.**007**	0.001
Target attire ^∗^ participant sex	1.11	1,6043.64	0.292	<0.001	2.050	1,5521.28	0.152	<0.001
Target sex ^∗^ others attire	1.52	1,6045.69	0.218	<0.001	0.11	1,5561.10	0.741	<0.001
Target sex ^∗^ participant sex	0.74	1,540.26	0.391	0.001	0.05	1,489.31	0.815	<0.001
Others attire ^∗^ participant sex	0.08	1,6039.05	0.778	<0.001	0.32	1,5560.11	0.571	<0.001
Target attire ^∗^ target sex ^∗^ others attire	2.65	1,6060.65	0.104	<0.001	0.03	1,5528.28	0.863	<0.001
Target attire ^∗^ target sex ^∗^ participant sex	7.24	1,6039.18	**0**.**007**	0.001	1.67	1,5521.30	0.196	<0.001
Target attire ^∗^ others attire ^∗^ participant sex	0.88	1,6055.73	0.349	<0.001	0.001	1,5528.55	0.972	<0.001
Target sex ^∗^ others attire ^∗^ participant sex	1.90	1,6040.29	0.168	<0.001	1.47	1,5559.12	0.225	<0.001
Target attire ^∗^ target sex ^∗^ others attire ^∗^ participant sex	1.53	1,6055.86	0.216	<0.001	0.98	1,5528.10	0.323	<0.001


*AIC, Akaike Information Criterion.*

**FIGURE 6:**
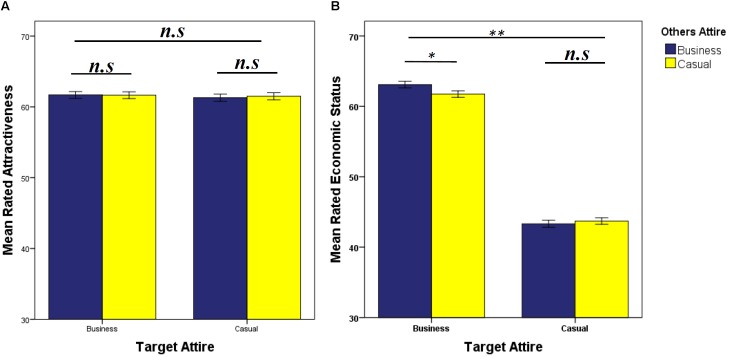
Data are mean ratings (±1 SEM) for **(A)** attractiveness and **(B)** economic status, split by other attire (business/casual) and target attire (business/casual). ^∗^*P* < 0.05, ^∗∗^*P* < 0.01, determined by *post hoc* least significance difference tests.

**FIGURE 7:**
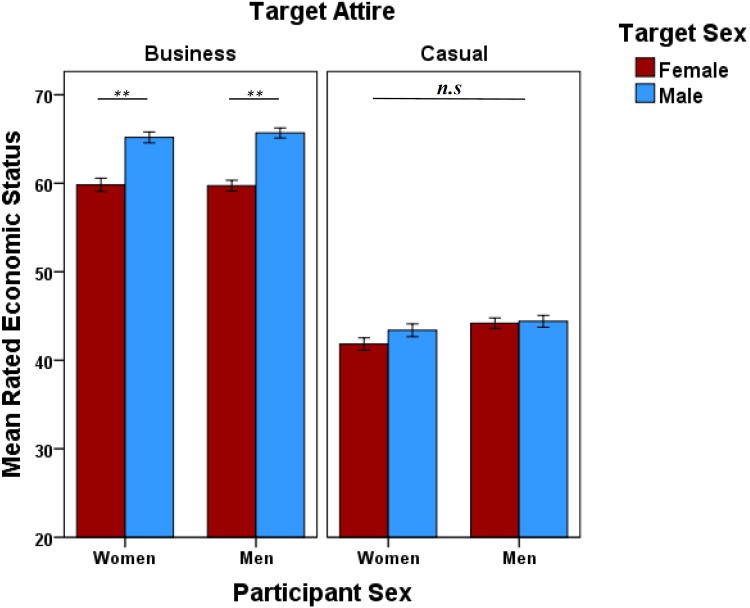
Data are mean economic status ratings (±1 SEM) split by participant sex (women and men), target attire (business/casual), and target sex (male/female). ^∗∗^*P* < 0.01, determined by *post hoc* least significance difference.

There were no significant main effects or interactions involving Other Attire on rated attractiveness (Table 3a and Figure 6A). However, there was a significant Other Attire × Target Attire interaction for ratings of economic status (Table 3b and Figure 6B). Targets in business attire presented alongside “others” in business attire were rated higher in economic status than when presented alongside “others” in casual attire (Figure 6B), which did not vary with Target Sex (Table 3b). A significant Target Sex × Target Attire interaction reflected men in business attire were rated significantly higher for economic status than women in business attire (Table 3b and Figure 7). Ratings of males and females in casual attire did not differ significantly (Figure 7). There were no significant main effects or interactions involving participant sex (Table 3b). For additional analyses see Supplementary Tables S4, S5.

### Discussion

As predicted, economic status ratings were higher when male and female targets were presented in business than casual attire. Additionally, male and female economic status ratings increased when presented alongside others in business attire. In contrast to Study 1, target females received lower attractiveness ratings in business than casual attire, which was driven by men’s ratings. The reduction in female attractiveness ratings when presented in business attire is consistent with previous research reporting high status women were judged negatively, less attractive, and less approachable than lower status women (Bassett et al., 1979; Forsythe, 1990; Heilman et al., 2004; Lavin A. et al., 2009). We also report female economic status was rated lower than the men they were presented alongside, indicating that perceptions of women’s economic status are influenced by the men they are presented with (Gouda-Vossos et al., 2016). Interestingly, the effects of economic status and sex disappeared when target males and females were presented in casual attire, so that sex differences in perceptions of status were specific to status-related social cues.

Our predictions regarding positive associations between attractiveness and economic status in male targets were supported, but only in males presented in business and not casual attire. Unexpectedly, we found positive associations between attractiveness and economic status ratings for women in both types of attire whereas previous research reported strong associations between economic status and attractiveness in males, with mixed results in women (Townsend and Levy, 1990; Hanson et al., 1991; Shuler and McCord, 2010). Our findings suggest that the influence of status-related clothing on judgments of attractiveness among women and men may be less robust than previously reported.

## General Discussion

A “*Wall Street*” article discussing attire and business practices stated that “traditional business dress is seen as a uniform…it simplifies decision making and makes hierarchies easy to read.” (Binkley, 2008). Our findings reinforce this sentiment, as participants made clear distinctions in physical attractiveness and economic status judgments based on clothing. We also report that women’s economic status is judged relative to and lower than the men they are presented alongside. These sex differences in judgments of economic status disappeared when target males and females were presented in casual attire, demonstrating that judgments of women and men’s economic status are most influenced by traditionally masculine clothing.

Status seeking is positively associated with men’s mating and reproductive success (Betzig, 1986; Hopcroft, 2006) with high economic status associated more with ideals surrounding maleness and masculinity than femininity (Akerlof and Kranton, 2000). Past studies have found that, regardless of sex, group members expressing masculine gender roles or dress in masculine attire are more likely to emerge as leaders and are judged as more forceful and aggressive than those expressing outwardly feminine characteristics (Goktepe and Schneier, 1989; Forsythe, 1990). A limitation of the current study was that we did not compare the effects within same sex groups. In our previous study (Gouda-Vossos et al., 2016), we reported that the economic status of target males was judged to be highest when presented with another man. High status men form same-sex alliances and partnerships (Von Rueden et al., 2010; Von Rueden, 2011), and the presence of two men not obviously in conflict may give the appearance that targets were forming same-sex partnerships. This was not the case when target females were presented alongside another female, as economic status was rated lower than when female targets were presented alone (Gouda-Vossos et al., 2016). In order to fully understand how men and women’s economic status are perceived within various group dynamics, and if economic status is truly judged within a masculine hierarchy, comparisons within same-sex groups would be a worthwhile extension of the current research.

In male dominated social environments characterized by defined hierarchies (Anderson et al., 2001; Schmid Mast, 2004) business attire is associated with more masculine and socially dominant attributes (Forsythe, 1990). Without clothing that clearly communicates economic status, it may be difficult for people to judge where others fall within a hierarchy, which may be why economic status ratings were more neutral when targets were presented in casual attire. It was also unsurprising that female attractiveness was rated lower when presented in business attire. However, this directly contradicts studies reporting no negative influence on female physical attractiveness when presented with high status cues such as cars (Brase and Richmond, 2004) or luxury apartments (Dunn and Hill, 2014). This suggests that judgments of female attractiveness are more likely to vary when women are presented as being of higher status rather than alongside high status cues. It could be argued that participants did not believe that the women in the study actually own the high-status cues (i.e., cars, luxury apartments, etc.); with attire being a more convincing indicator of earned status. By presenting target females as high status individuals, it may communicate economic independence and decrease the attractiveness of female targets to men.

The current study also found positive associations between attractiveness and economic status among male and female targets, except for males presented in casual attire. Judgments of men’s economic status and physical attractiveness are strongly positively correlated (Townsend and Levy, 1990; Hanson et al., 1991; Shuler and McCord, 2010) with competence, financial worth, and credibility being more consistently associated with men in business than casual attire (Bassett et al., 1979; Morris et al., 1996; Lavin A. M. et al., 2009). However, even subtle differences in attire within male-dominated business environments can lead to negative criticisms and attitudes toward men. For instance, men are perceived to be less confident, successful, and having lower salaries when presented in “off the peg” suits as opposed to “tailored suits” (Howlett et al., 2013). Further, men experience greater verbal harassment when presented in non-traditional business attire (i.e., business casual) than when presented in business attire (Kwantes et al., 2011). It could be argued that the economic status of more attractive male targets in casual attire was penalized in the current study, demonstrating how culturally malleable cues of status interplays with male attractiveness, possibly influencing women’s mate preferences.

## Conclusion

Maestripieri et al. (2017) marshaled a comprehensive review on financial and prosocial biases and concluded that attractive individuals, especially women, were more likely to attain financial benefits and better treatment than their less attractive counterparts. The results of the current studies demonstrated that high status individuals, especially men, receive more favorable judgments relating to mate choice (i.e., attractiveness). However, whether this leads to better treatment or increased financial gain remains to be fully explored. Ostensibly, men and women both benefit from being highly attractive or high status, however, this benefit is not distributed equally. Although this is consistent with ideals surrounding the asymmetrical importance of status in males and physical attractiveness in women within mating contexts, the results of the current studies reflect how this may lead to unfair judgments and, possibly, unfair treatment of both men and women.

## Author Contributions

AG-V, RB, and BD contributed conception and design of the study. AG-V carried out studies and organized the database. AG-V, RB, and BD performed the statistical analysis. AG-V wrote the first draft of the manuscript. AG-V, RB, and BD wrote sections of the manuscript. All authors contributed to manuscript revision, read, and approved the submitted version.

## Conflict of Interest Statement

The authors declare that the research was conducted in the absence of any commercial or financial relationships that could be construed as a potential conflict of interest.

## Footnotes

1 www.peopleimages.com

2 www.peopleimages.com

3 www.socialsci.com

## Supplementary Material

MATERIAL S1 Example images used for targets presented alone and with opposite sex others.

MATERIAL S2 *Ad Hoc* MLMs of rated attractiveness of male and female models by men and women, factoring in ‘target attire’.

MATERIAL S3 *Ad Hoc* MLMs of rated economic status of male and female models by men and women, factoring in ‘target attire’.

MATERIAL S4 Data for groups study 1 separated by target sex and measure (attractiveness, earnings).

MATERIAL S5 Data for attire study 2 separated by measure (attractiveness, earnings).
