# Supplementary material for: The Interplay Between Economic Status and Attractiveness, and the Importance of Attire in Mate Choice Judgments
Source: Front Psychol. 2019 Mar 21;10:462. doi: 10.3389/fpsyg.2019.00462 (PMC6437035; doi:10.3389/fpsyg.2019.00462)
Supplement: MATERIAL S1 — Example images used for targets presented alone and with opposite sex others. [file Table_1.docx]

| **Female Targets** | **Alone** | **+ 1 Opposite Sex Other** | **+ 2 Opposite Sex Other** | **+ 4 Opposite Sex Other** |
| --- | --- | --- | --- | --- |
|  | 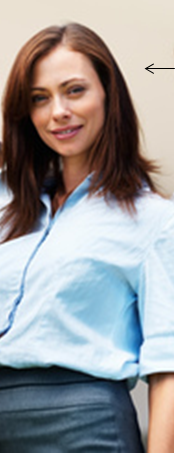 | 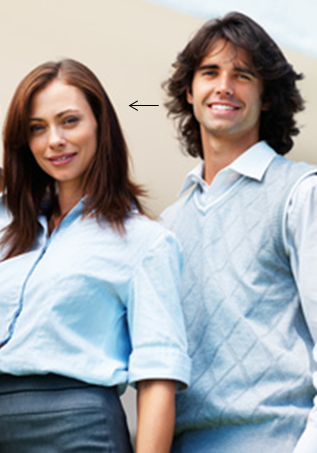 | 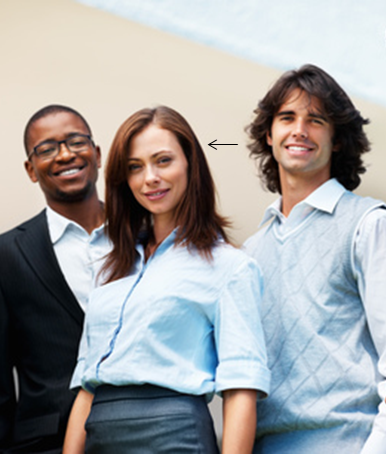 | 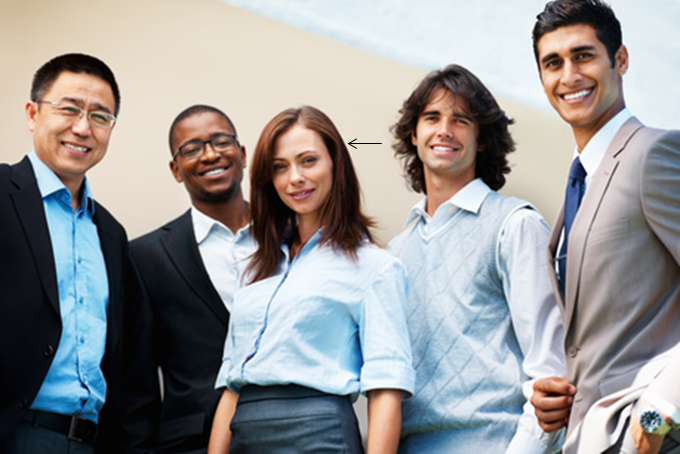 |
| **Male Targets** | 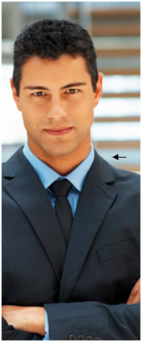 | 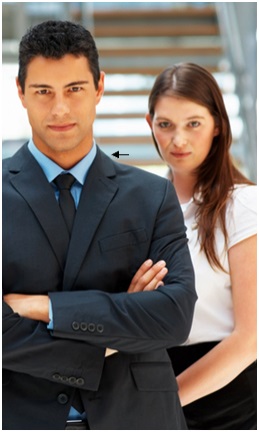 | 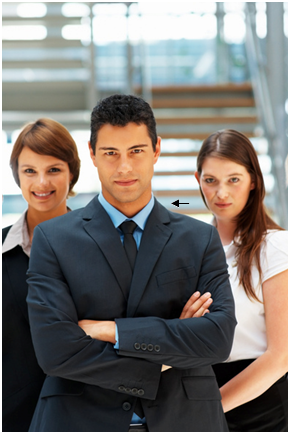 | 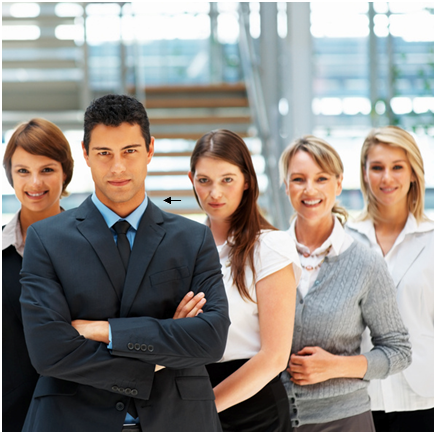 |

ESM 1: Example images used for targets presented alone and with opposite sex others
