# Supplementary material for: The Interplay Between Economic Status and Attractiveness, and the Importance of Attire in Mate Choice Judgments
Source: Front Psychol. 2019 Mar 21;10:462. doi: 10.3389/fpsyg.2019.00462 (PMC6437035; doi:10.3389/fpsyg.2019.00462)
Supplement: MATERIAL S2 — Ad Hoc MLMs of rated attractiveness of male and female models by men and women, factoring in ‘target attire’. [file Table_2.docx]

### **Electronic Supplementary Material 1**

### **Table S1**

***Ad Hoc* MLMs of rated attractiveness of male and female models by men and women, factoring in ‘target attire’.**

|  | **Target Female Attractiveness** | | | |  | **Target Male Attractiveness** | | | |
| --- | --- | --- | --- | --- | --- | --- | --- | --- | --- |
| **AIC** | **26768.358** | | | |  | **23657.820** | | | |
|  | ***d.f.*** | **F** | ***p*** | ***Ƞp^2^*** |  | ***d.f.*** | **F** | ***P*** | ***Ƞp^2^*** |
| **Group Size** | 3,2952.477 | 1.937 | 0.121 | 0.00196 |  | 3,2525.324 | 0.091 | 0.965 | 0.00011 |
| **Participant Sex** | 1,2962.516 | 2.982 | 0.084 | 0.00101 |  | 1,2554.580 | 0.916 | 0.339 | 0.00036 |
| **Target Attire** | 1,2962.516 | 68.133 | **< 0.001** | 0.02248 |  | 1,2554.580 | 0.851 | 0.356 | 0.00033 |
| **Group Size* Participant Sex** | 3,2952.477 | 0.234 | 0.873 | 0.00024 |  | 3,2525.324 | 0.935 | 0.423 | 0.00111 |
| **Group Size* Target Attire** | 3,2952.477 | 0.506 | 0.678 | 0.00051 |  | 3,2525.324 | 0.837 | 0.473 | 0.00099 |
| **Participant Sex * Target Attire** | 1,2962.516 | 1.551 | 0.213 | 0.00052 |  | 1,2554.580 | 0.741 | 0.390 | 0.00029 |
| **Group Size * Participant Sex * Target Attire** | 3,2952.477 | 1.039 | 0.374 | 0.00105 |  | 3,2525.324 | 0.547 | 0.650 | 0.00065 |
