# Supplementary material for: The Interplay Between Economic Status and Attractiveness, and the Importance of Attire in Mate Choice Judgments
Source: Front Psychol. 2019 Mar 21;10:462. doi: 10.3389/fpsyg.2019.00462 (PMC6437035; doi:10.3389/fpsyg.2019.00462)
Supplement: MATERIAL S3 — Ad Hoc MLMs of rated economic status of male and female models by men and women, factoring in ‘target attire’. [file Table_3.docx]

### **Electronic Supplementary Material 2**

***Ad Hoc* MLMs of rated economic status of male and female models by men and women, factoring in ‘target attire’.**

|  | **Target Female Economic Status** | | | |  | **Target Male Economic Status** | | | |
| --- | --- | --- | --- | --- | --- | --- | --- | --- | --- |
| **AIC** | **18405.626** | | | |  | **21434.759** | | | |
|  | ***d.f.*** | **F** | ***P*** | ***Ƞp^2^*** |  | ***d.f.*** | **F** | ***P*** | ***Ƞp^2^*** |
| **Group Size** | 3,2057.780 | 3.181 | **0.023** | 0.00462 |  | 3,1853.191 | 1.408 | 0.239 | 0.00227 |
| **Participant Sex** | 1,2075.065 | 2.509 | 0.113 | 0.00121 |  | 1,1876.782 | 2.156 | 0.142 | 0.00115 |
| **Target Attire** | 1,2075.065 | 356.538 | **< 0.001** | 0.14663 |  | 1,1876.782 | 533.833 | **< 0.001** | 0.22145 |
| **Group Size* Participant Sex** | 3,2057.780 | 0.056 | 0.982 | 0.00008 |  | 3,1853.191 | 0.280 | 0.840 | 0.00045 |
| **Group Size* Target Attire** | 3,2057.780 | 6.369 | **< 0.001** | 0.00920 |  | 3,1853.191 | 0.459 | 0.711 | 0.00074 |
| **Participant Sex * Target Attire** | 1,2075.065 | 18.681 | **< 0.001** | 0.00892 |  | 1,1876.782 | 0.686 | 0.408 | 0.00037 |
| **Group Size * Participant Sex * Target Attire** | 3,2057.780 | 0.692 | 0.557 | 0.00101 |  | 3,1853.191 | 0.489 | 0.690 | 0.00079 |
